# Comprehensive analysis of molecular mechanism and a novel prognostic signature based on small nuclear RNA biomarkers in gastric cancer patients

**DOI:** 10.1515/med-2022-0493

**Published:** 2022-05-30

**Authors:** Ziyu Liang, Dongxing Su, Kang Liu, Haixing Jiang

**Affiliations:** Department of Gastroenterology, The Third Affiliated Hospital of Guangxi Medical University, Nanning, 530000, Guangxi Zhuang Autonomous Region, People’s Republic of China; Department of Radiation Oncology, The First Affiliated Hospital of Guangxi Medical University, Nanning, 530021, Guangxi Zhuang Autonomous Region, People’s Republic of China; Department of Gastroenterology, The First Affiliated Hospital of Guangxi Medical University, Shuang Yong Road 6, Nanning, 530021, Guangxi Zhuang Autonomous Region, People’s Republic of China

**Keywords:** small nuclear RNA, molecular mechanism, gastric cancer, overall survival, The Cancer Genome Atlas

## Abstract

Small nuclear RNAs (snRNAs) are rarely reported in cancer. This study is based on The Cancer Genome Atlas genome-wide data set to explore the prognostic value and molecular mechanism of snRNAs in gastric cancer (GC). Gene ontology, Kyoto Encyclopedia of Genes and Genomes, and gene set enrichment analysis were used to explore the molecular mechanism of snRNAs. A total of 351 patients were included in the survival analysis, and 14 prognostic snRNAs were identified using multivariate survival analysis. We constructed a prognostic signature containing nine snRNAs, which can signally classify patients into high- and low-risk phenotypes (adjusted *P* < 0.0001, hazard ratio = 2.671, 95% confidence interval = 1.850–3.858). Combining the molecular mechanisms obtained by the three functional enrichment approaches, we concluded that this prognostic signature snRNAs participated in classical tumor-related signaling pathways, including Notch, PI3K, toll-like receptor, etc.; cell adhesion; cell cycle; cell proliferation; and other biological processes that affect the biological phenotype of cancer cells. We also found significant downregulation of the abundance of immune cell infiltrates and immune microenvironment scores for high-risk phenotypes of GC patients. In conclusion, this study has identified 14 prognostic snRNAs signally associated with GC overall survival and also constructed a novel prognostic signature containing nine prognostic snRNAs.

## 1 Introduction

Gastric cancer (GC) is a common type of cancer worldwide; 5-year relative survival rate is about 20% [1,2]. The main pathological type was gastric adenocarcinoma. The prognosis of GC is directly related to the stage at the time of diagnosis. To this day, surgery is still the major strategy for GC [3,4]. However, due to the low diagnosis rate of GC at the early stage, the survival rate is only about 10% [5,6]. Most GC patients are in an advanced stage at the time of diagnosis, and the 5-year survival rate is about 7–34% [1]. Hence, this is an impendency requirement to develop efficient biomarkers for GC diagnosis and prognostic assessment [7,8]. The study has reported that normally small nuclear RNA (snRNA) does not exist free, but combines with protein to form a complex and becomes small nuclear ribonucleoprotein particles (snRNPs) [9,10]. It has been confirmed that snRNAs do not participate in protein synthesis, and their main function is to play an indispensable role in RNA processing. The protein part of snRNA has nuclease and ligase activities, which can cut transcription at the intron–exon junction and connect the two free ends [9,11]. snRNA participates in the construction of nucleoprotein complexes and performs splicing functions by pairing bases with target site RNA or snRNA [12,13]. At the same time, it has also been reported that snRNA may be involved in gene activation and the processing of rRNA precursors. As a component of chromatin and nuclear structure, snRNA may play an important role in maintaining its special structure and may be involved in chromatin replication and transcription [11,14]. With the rapid development of high-throughput sequencing technology, more and more non-coding RNAs have been discovered, and their molecular mechanisms have been continuously verified and elucidated. By reviewing the literature, we note that snRNAs have been mentioned in a large number of non-coding RNA-related studies, but few studies have specifically focused on snRNAs, especially in cancer studies. To make up for the research gap of snRNAs in cancers. In this study, the clinical outcome and molecular mechanism of snRNAs in GC were comprehensively explored using The Cancer Genome Atlas (TCGA) genome-wide RNA sequencing data.

## 2 Material and methods

### 2.1 Data download and normalization

The RNA sequencing data of the GC cohort were downloaded from TCGA Data Portal (https://portal.gdc.cancer.gov) [15,16]. EdgeR was used to normalize raw RNA sequencing data [17]. We obtained RNA sequencing data from 407 samples in total, including 32 paracancerous tissue samples and 375 tumor tissue samples. We excluded 7 patients with missing survival information and 17 patients with overall survival (OS) time of zero. Finally, 351 GC patients were participated in the final survival analysis. We extracted 1,872 snRNAs from the RNA sequencing data set. After edgeR normalization, we filter out snRNAs with a mean value of less than 1 and finally got 554 snRNAs for inclusion in the final survival analysis. As the authors did not perform any animal or human experiments in this study, ethics committee approval was not required.

### 2.2 Survival analysis and construction of snRNA signature

We screened the prognostic snRNAs using a multivariate Cox proportional hazards regression model, and the step function was used to construct a prognostic signature model for these prognostic snRNAs. After screening by a step function, we will accumulate bonus points for multiple snRNAs and get a comprehensive score. A comprehensive risk assessment of the patient was conducted to separate high- and low-risk GC patients: Risk score = expression of snRNA1 × *β*1 + expression of snRNA2 × *β*2 + … expression of snRNAn × *βn* [18,19,20,21]. Survival receiver operating characteristic (survivalROC) analysis can be applied to assess the prognostic accuracy of this signature. The nomogram is based on the prognostic signature and routine clinical information for scoring in individualized prognostic analysis.

### 2.3 Investigation of the molecular mechanism of snRNA prognostic signature

To investigate the molecular mechanisms of this snRNA signature, we employed three different functional enrichment analysis methods for molecular mechanism exploration.

First, we obtained a protein-coding gene data set from RNA-sequencing data, and the data normalization method was also carried out using edgeR. Screening of snRNA co-expressed genes by Pearson correlation coefficient (*r*), |*r*| >0.4, and *P* < 0.05 were considered as snRNA co-expressed genes. Gene Ontology (GO) and Kyoto Encyclopedia of Genes and Genomes (KEGG) were performed using the DAVID V6.8 (https://david.ncifcrf.gov/home.jsp) online analysis tool [22,23,24]. Second, we also use clusterProfiler to perform gene set enrichment analysis (GSEA) in R platform [25,26]. Finally, we also screened differentially expressed genes (DEGs) for high- and low-risk group patients in the R platform by the edgeR package. Functional enrichment analysis of these DEGs was subsequently performed to screen out the molecular mechanisms underlying this prognostic signature. The criteria for identifying differentially expressed genes are as follows: |log2 fold change (FC)| >1, *P* < 0.05, and false discovery rate (FDR) <0.05. We also performed a co-expression network analysis of DEGs using weighted gene co-expression network analysis (WGCNA) to facilitate the identification of hub DEGs [27,28].

### 2.4 Immunomicroenvironment and ssGSEA analysis

Immune microenvironment scoring is performed in the R platform by the Estimation of STromal and Immune cells in MAlignant Tumours using Expression data’ (ESTIMATE) package [29]. Single sample gene set enrichment analysis (ssGSEA) analysis was performed in the R platform with gene set variation analysis packages [30,31].

### 2.5 Statistical analysis

The co-expressed genes of snRNAs were determined using Pearson correlation coefficient *r* assessed using an independent sample *t* test. Kaplan–Meier analysis was applied to univariate survival analysis, and Cox proportional hazards regression model was used for multivariate survival analysis. Statistical analysis was performed using the SPSS version22 or R version 4.0.2. Statistical significance was determined at *P* < 0.05.

## 3 Results

### 3.1 Comprehensive survival analysis and construction of snRNA signature

**Figure 1:**
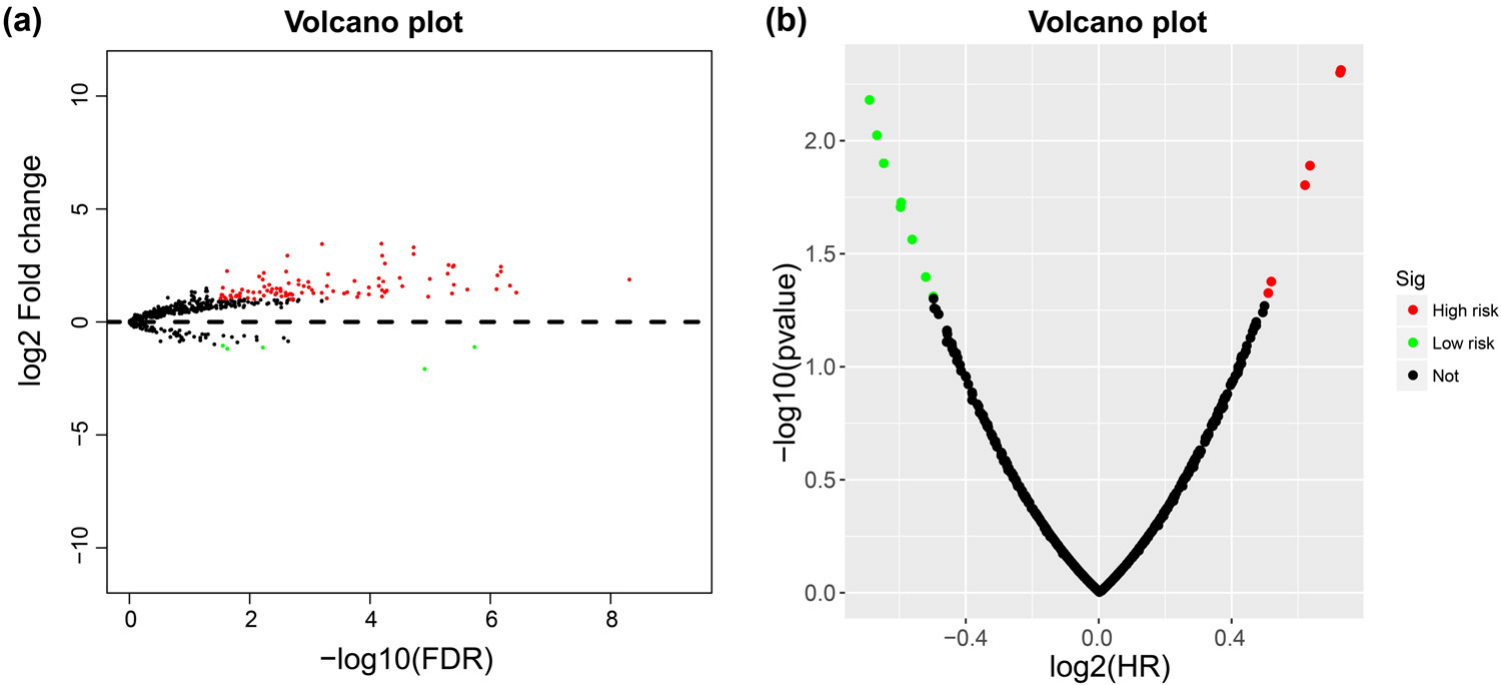
Volcano plot of fold change and survival analysis of snRNAs in GC: (a) volcano plot of the fold change of snRNAs in GC; (b) volcano plot of prognosis analysis of snRNAs in GC.

**Figure 2:**
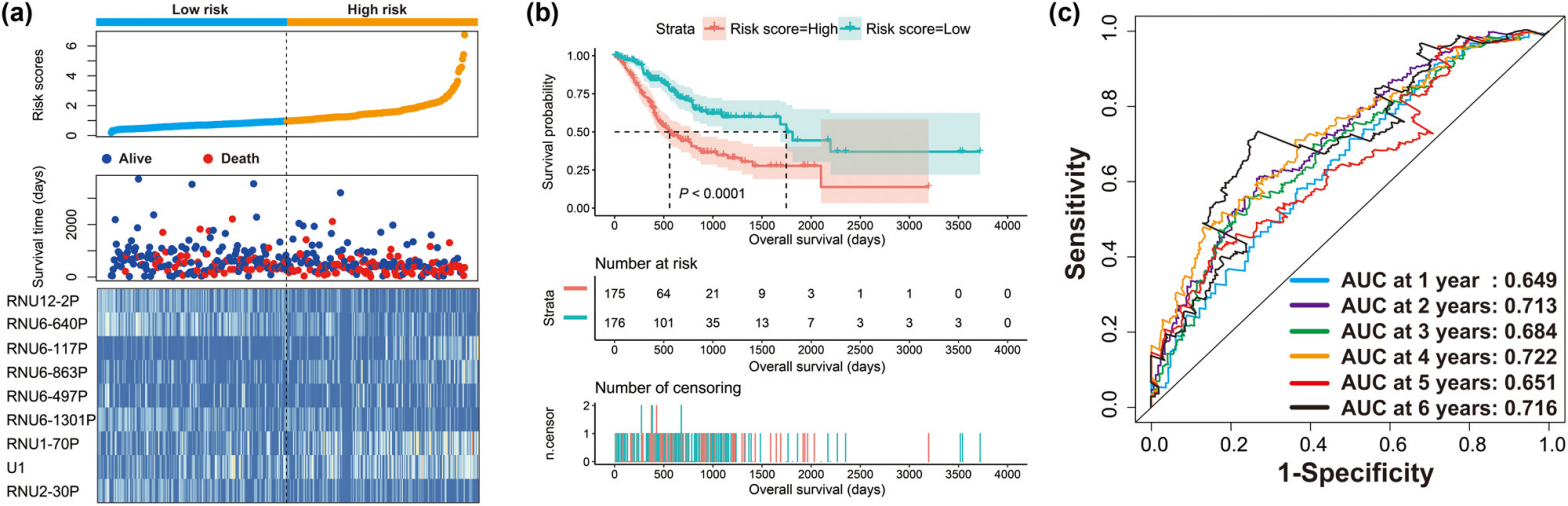
Prognostic value of this prognostic signature containing nine snRNAs: (a) risk score and survival time distribution plot; (b) Kaplan–Meier curve of the risk score in GC; (c) SurvivalROC curve of the risk score in GC.

**Figure 3:**
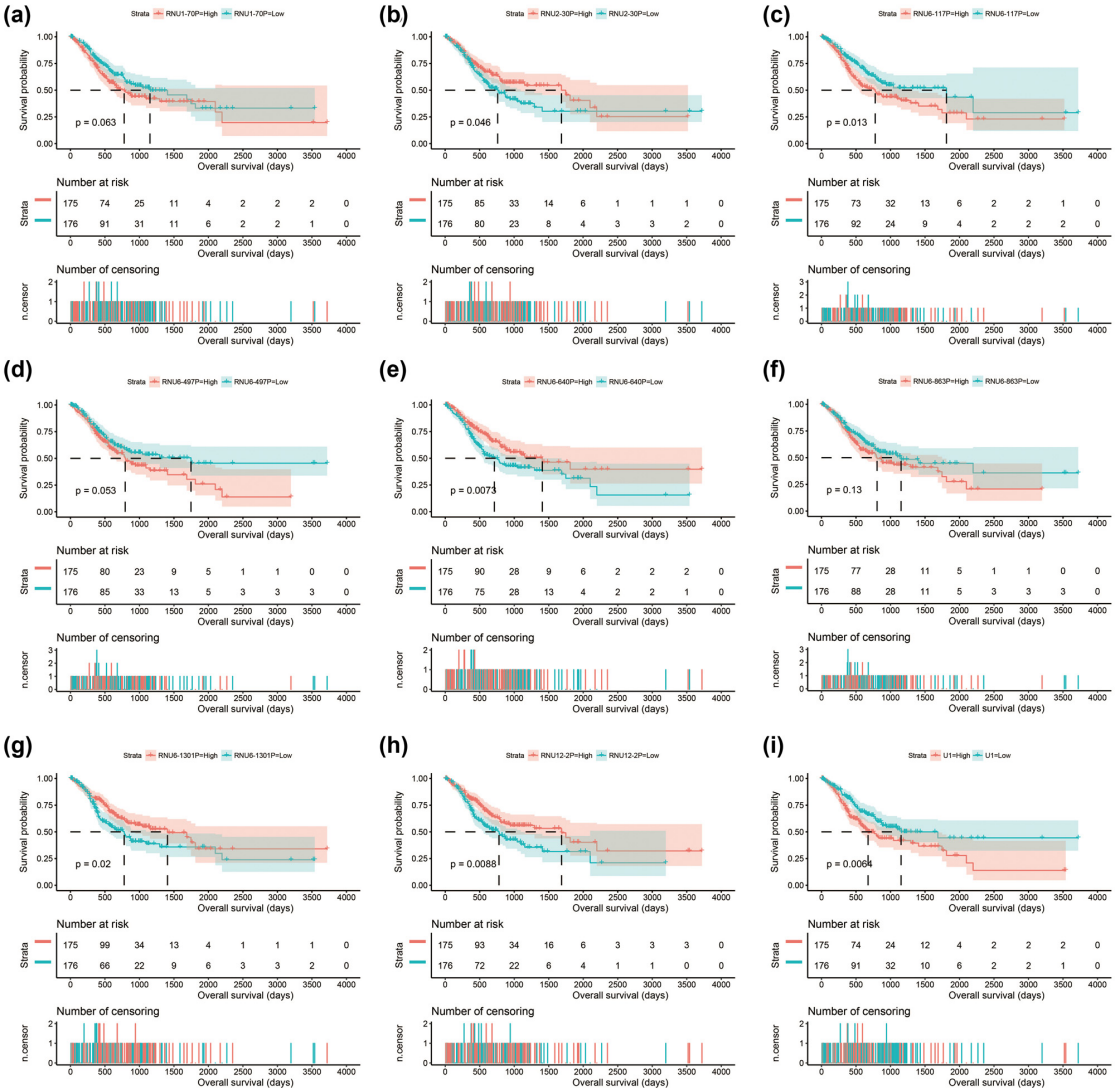
Kaplan–Meier curve of this prognostic signature containing nine snRNAs in GC: (a) RUN1–70P; (b) RUN2–30P; (c) RUN6–117P; (d) RUN6–497P; (e) RUN6–640P; (f) RUN6–863P; (g) RUN6–1301P; (h) RUN12–2P; and (i) U1.

**Figure 4:**
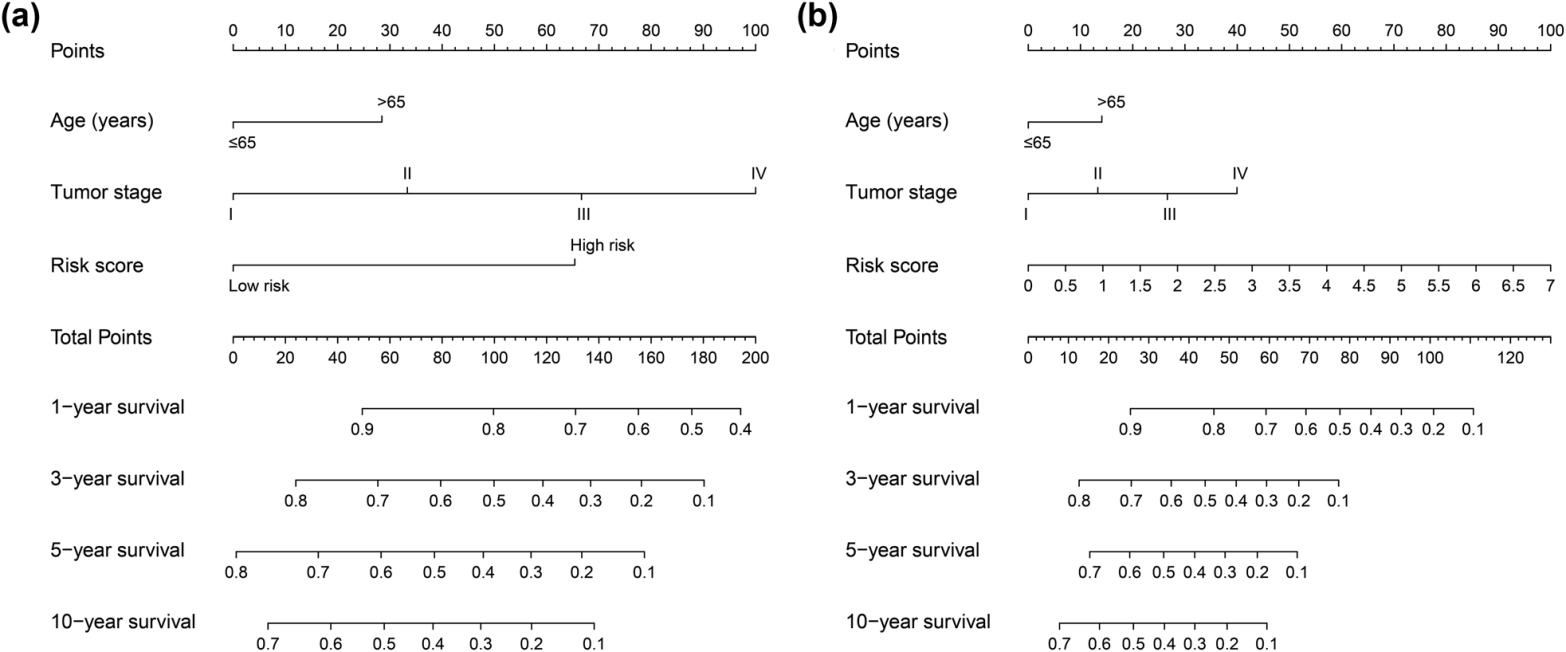
Nomogram for risk score in GC OS: (a) nomogram for low- and high-risk score phenotypes in GC OS; (b) nomogram for risk score in GC OS.

**Figure S1:**
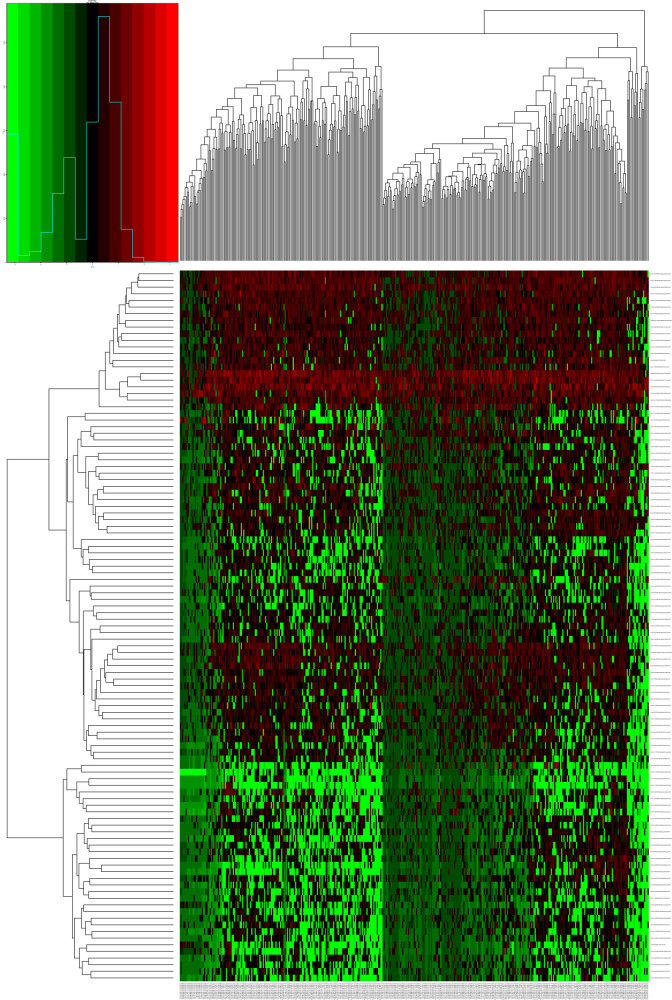
Heat map of differentially expressed snRNAs between tumor and paracancer tissues of gastric cancer.

By using edgeR to compare the expression abundance of snRNA between cancer and adjacent tissues in the TCGA GC queue, we obtained a total of 107 differentially expressed snRNAs. Among them, 5 snRNAs were significantly downregulated and 102 snRNAs were signally upregulated (Figure 1a, Figure S1 and Table S1). All snRNA differentially expressed fold changes are summarized in Table S1. The most signally downregulated snRNA was RNU1-70P (ENSG00000199488, log2FC = ‒2.09, *P* < 0.0001, and FDR < 0.0001), and the most signally upregulated snRNA was RNU6-438P (ENSG00000202431, log2FC = 3.460, *P* < 0.0001, and FDR <0.0001). Baseline parameters of patients in the TCGA GC cohort are summarized in Table S2. We observed that age (log-rank *P* = 0.011) and tumor stage (log-rank *P* < 0.0001) were signally associated with GC OS (Table S2), and these two clinical parameters were included in the subsequent multivariate Cox proportional hazards regression model. By performing survival analysis on all snRNAs, we observed that 14 snRNAs were signally related to GC OS in the TCGA cohort (Figure 1b). Among them, the most significant is RNU6–117P (ENSG00000202285, adjusted *P* = 0.0049, hazard ratio (HR) = 1.654, 95% confidence interval [CI] = 1.165–2.348, Table S3). In subsequent prognostic signature construction, we screened out an optimal combination containing nine prognostic snRNAs, including RNU12–2P (ENSG00000201659), RNU6–640P (ENSG00000200563), RNU6–117P (ENSG00000202285), RNU6–863P (ENSG00000251798), RNU6–497P (ENSG00000202186), RNU6–1301P (ENSG00000199594), RNU1–70P (ENSG00000199488), U1 (ENSG00000274428), and RNU2–30P (ENSG00000252018). We observed that this prognostic signature could divide GC patients into two groups with significant prognostic differences. Patients in the high-risk phenotype had a signally higher risk of death than those in the low-risk phenotype (adjusted *P* < 0.0001, HR = 2.671, 95% CI = 1.850–3.858; Figure 2a and b). SurvivalROC evaluation showed that the prediction precision of the snRNAs signature was highest at the time point of 4 years, and the area under the curve was 0.722 (Figure 2c). Using Kaplan–Meier univariate survival analysis, we observed that three snRNAs (RNU1–70P, RNU6–497P, and RNU6–863P) were not signally related to OS, but after multivariate adjustment, they were found to be significantly associated with GC OS (Figure 3a–i). Nomogram analysis of this prognostic signature also showed that risk score contributed the most to GC death compared to traditional clinical parameters (Figure 4a–b).

### 3.2 Investigation of the molecular mechanism of snRNA prognostic signature

**Figure 5:**
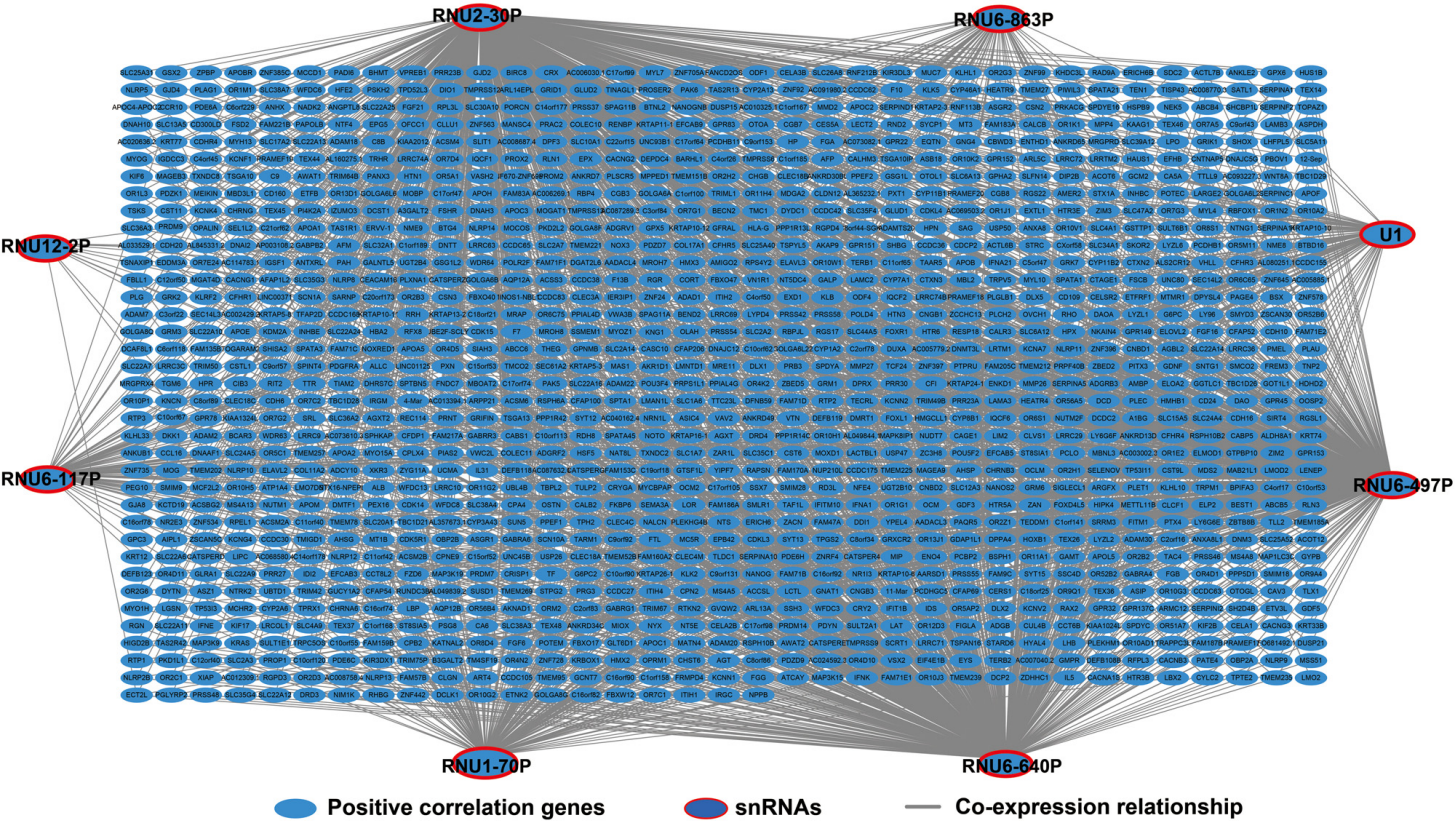
Interaction network of prognostic snRNAs and mRNA co-expression pairs.

**Figure 6:**
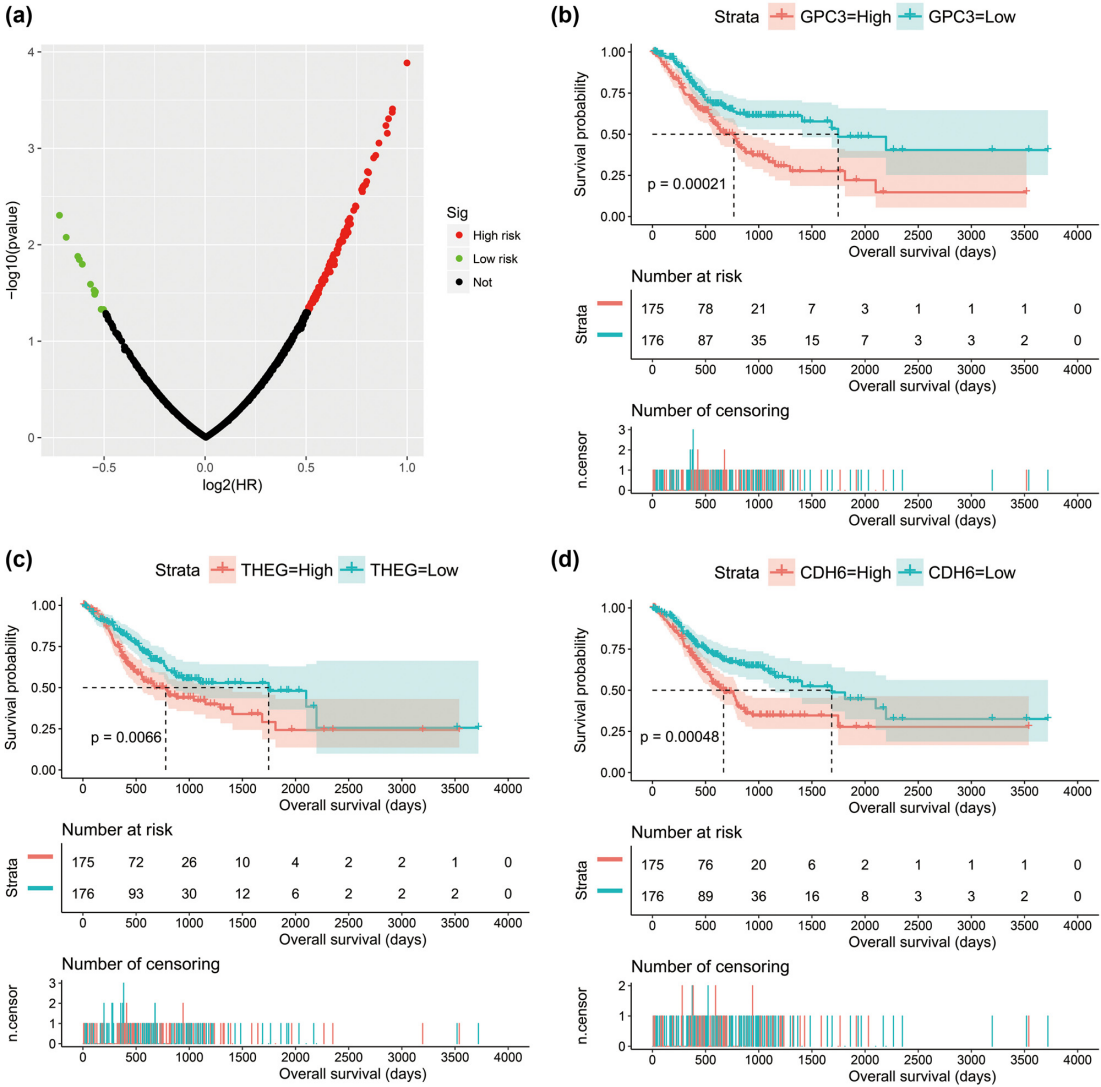
Survival analysis results of snRNA co-expressed genes: (a) volcano plot of survival analysis results; (b) Kaplan–Meier curve of glypican 3 (GPC3); (c) Kaplan–Meier curve of theg spermatid protein (THEG); and (d) Kaplan–Meier curve of cadherin 6 (CDH6).

By screening the co-expression-related genes of nine snRNAs, we got 2,481 snRNA–mRNA co-expression relationship pairs (Figure 5, Table S4). Our survival analysis of snRNA co-expressed genes observed that 122 protein-coding genes were significantly associated with GC OS (Figure 6a–d, Table S5). Functional enrichment investigation of these snRNA co-expressed genes revealed that they are involved in the following biological functions and pathway mechanisms: G-protein-coupled receptor activity, G-protein coupled receptor signaling pathway, retinoid metabolic process, regulation of Cdc42 protein signal transduction, cell differentiation, triglyceride catabolic process, and negative regulation of BMP signaling pathway (Table S6).

**Figure 7:**
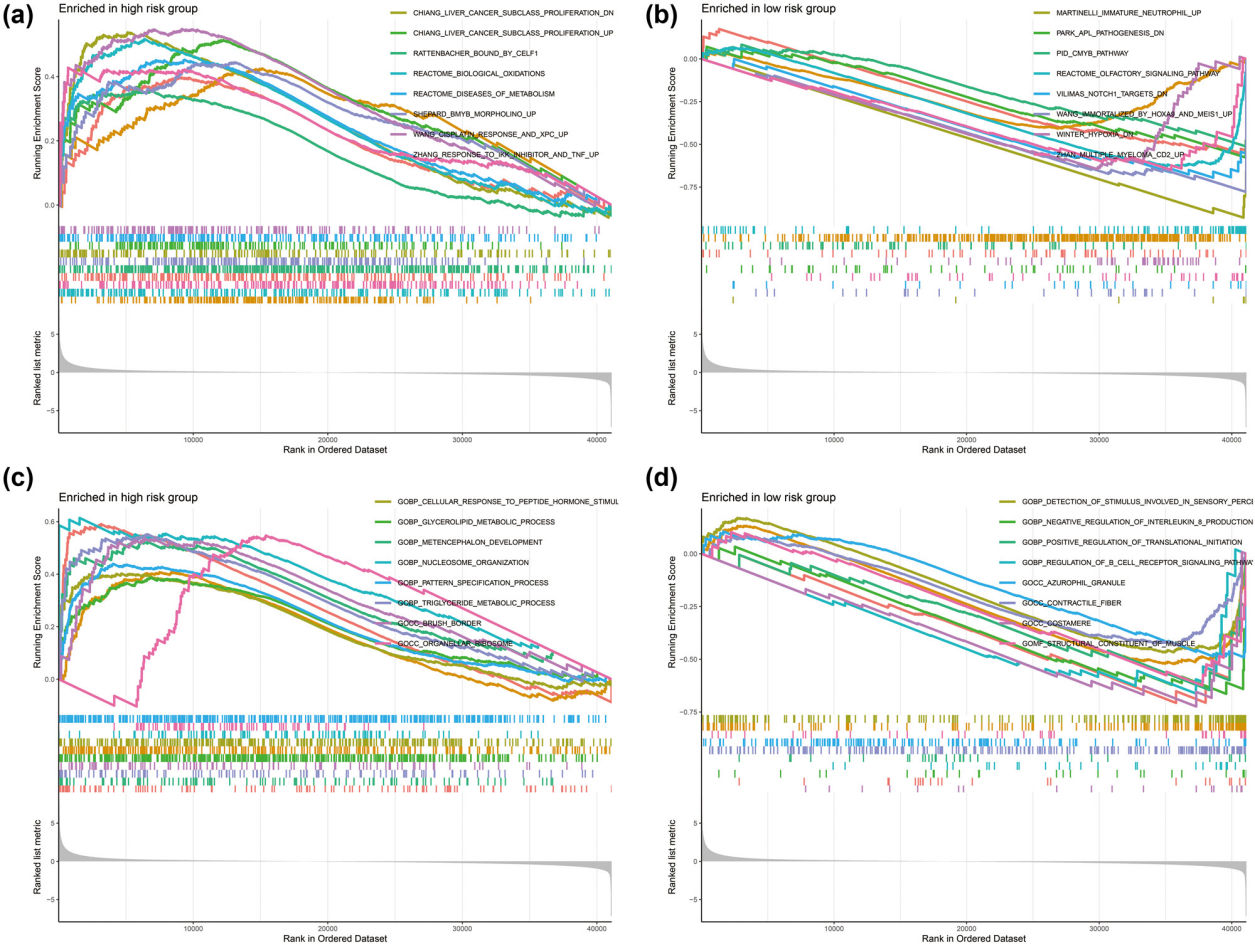
GSEA results between low- and high-risk score phenotypes in GC: (a) GSEA results in high-risk score group using the c2 reference gene set; (b) GSEA results in low-risk score group using the c2 reference gene set; (c) GSEA results in high-risk score group using the c5 reference gene set; and (d) GSEA results in low-risk score group using the c5 reference gene set.

GSEA for pathway investigation revealed that GC patients in high-risk group are signally different from low-risk group in the following pathways: silenced by tumor microenvironment, P73 pathway, toll like receptor (TLR) TLR1/TLR2 cascade, signal transducer and activator of transcription 3 (STAT3) targets up, integrin3 pathway, metastasis epithelial–mesenchymal transition up, transforming growth factor beta 1 signaling, mitogen-activated protein kinase kinase (MAP2K) and mitogen-activated protein kinase (MAPK) activation, epidermal growth factor receptor signaling 24 h up, apoptosis by cyclin dependent kinase inhibitor 1A via tumor protein P53 (TP53), GC early up, peroxisome proliferator-activated receptor signaling pathway, hypoxia inducible factor 1 subunit alpha pathway, oncogenic MAPK signaling, apoptosis via nuclear factor kappa-B (NFκB), Myc oncogenic signature, TP53 and tumor protein P63 (TP63) targets, cell cycle checkpoints, mechanistic target of rapamycin kinase (mTOR) 4pathway, cell cycle mitotic, and phosphatidylinositol 3-kinase (PI3K)/Akt signaling pathway (Figure 7a, Table S7). While the low-risk group was notably related to the following mechanisms: B-cell receptor signaling pathway, T-cell receptor pathway, PI3K cascade fibroblast growth factor receptor 2 (FGFR2), phospholipase-c-mediated cascade FGFR2, and interleukin 2 family signaling (Figure 7b, Table S7). GSEA for GO term investigation revealed that GC patients in the high-risk group are signally different from the low-risk group in the following biological processes: ncRNA processing, positive regulation of cell cycle, TLR signaling pathway, B-cell-mediated immunity regulation of receptor signaling pathway via STAT, regulation of Notch signaling pathway, cell–cell junction assembly, regulation of apoptotic signaling pathway, Notch signaling pathway, developmental cell growth, and regulation of cell–cell adhesion (Figure 7c, Table S8). While the low-risk group was significantly associated with the following mechanisms: B cell proliferation, mature B cell differentiation involved in immune response, 3′-UTR-mediated mRNA stabilization, T-cell receptor complex, regulation of B-cell receptor signaling pathway, and negative regulation of interleukin 8 production (Figure 7d, Table S8).

**Figure 8:**
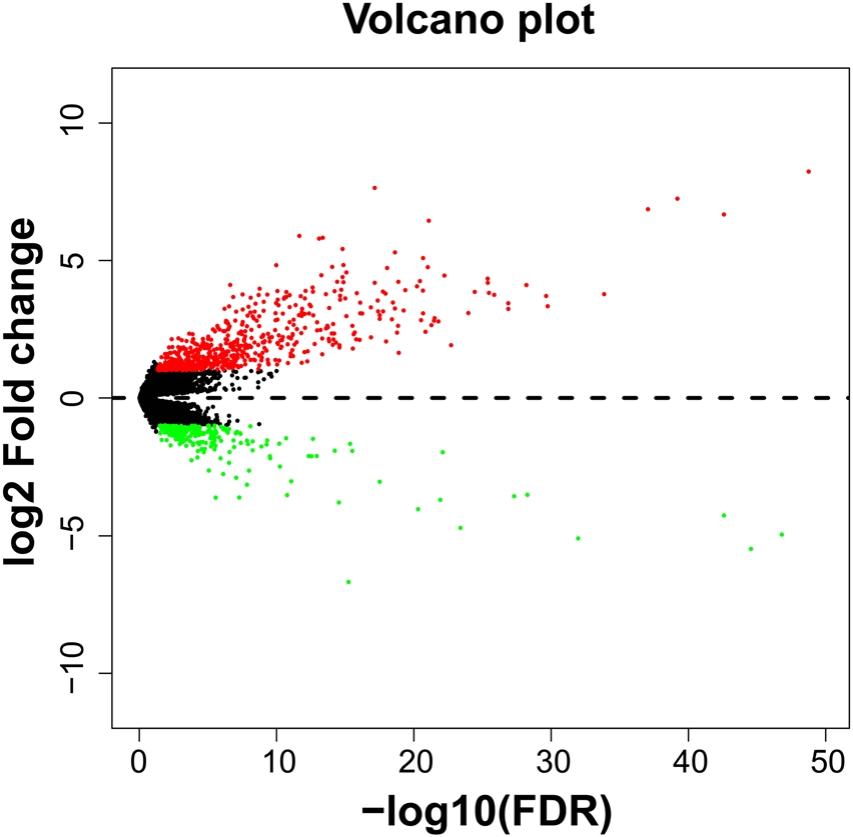
Volcano plot of DEGs between low- and high-risk score phenotypes in GC.

**Figure 9:**
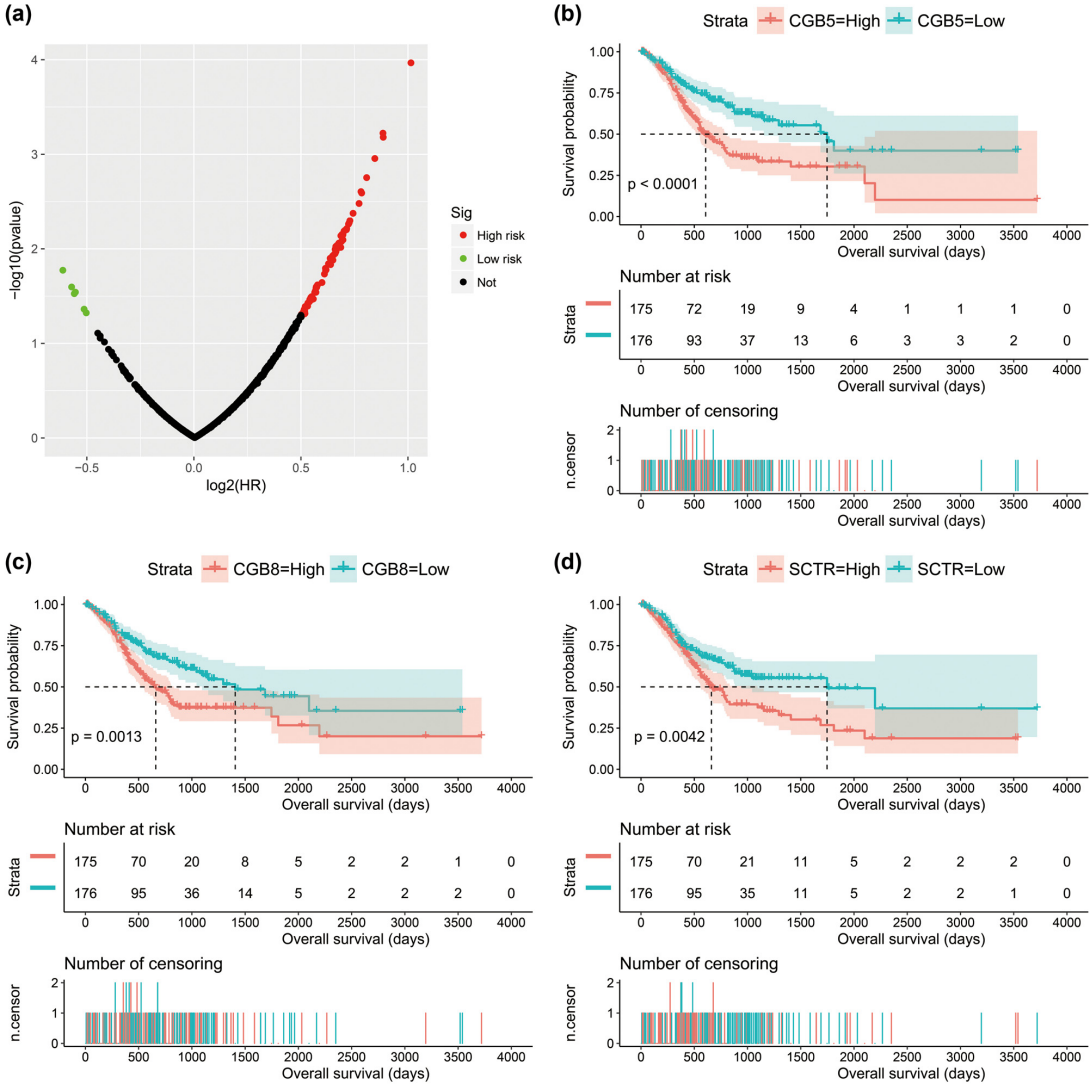
Survival analysis of DEGs between low- and high-risk score phenotypes in GC: (a) volcano plot of survival analysis results; (b) Kaplan–Meier curve of CGB5; (c) Kaplan–Meier curve of CBG8; and (d) Kaplan–Meier curve of SCTR.

**Figure 10:**
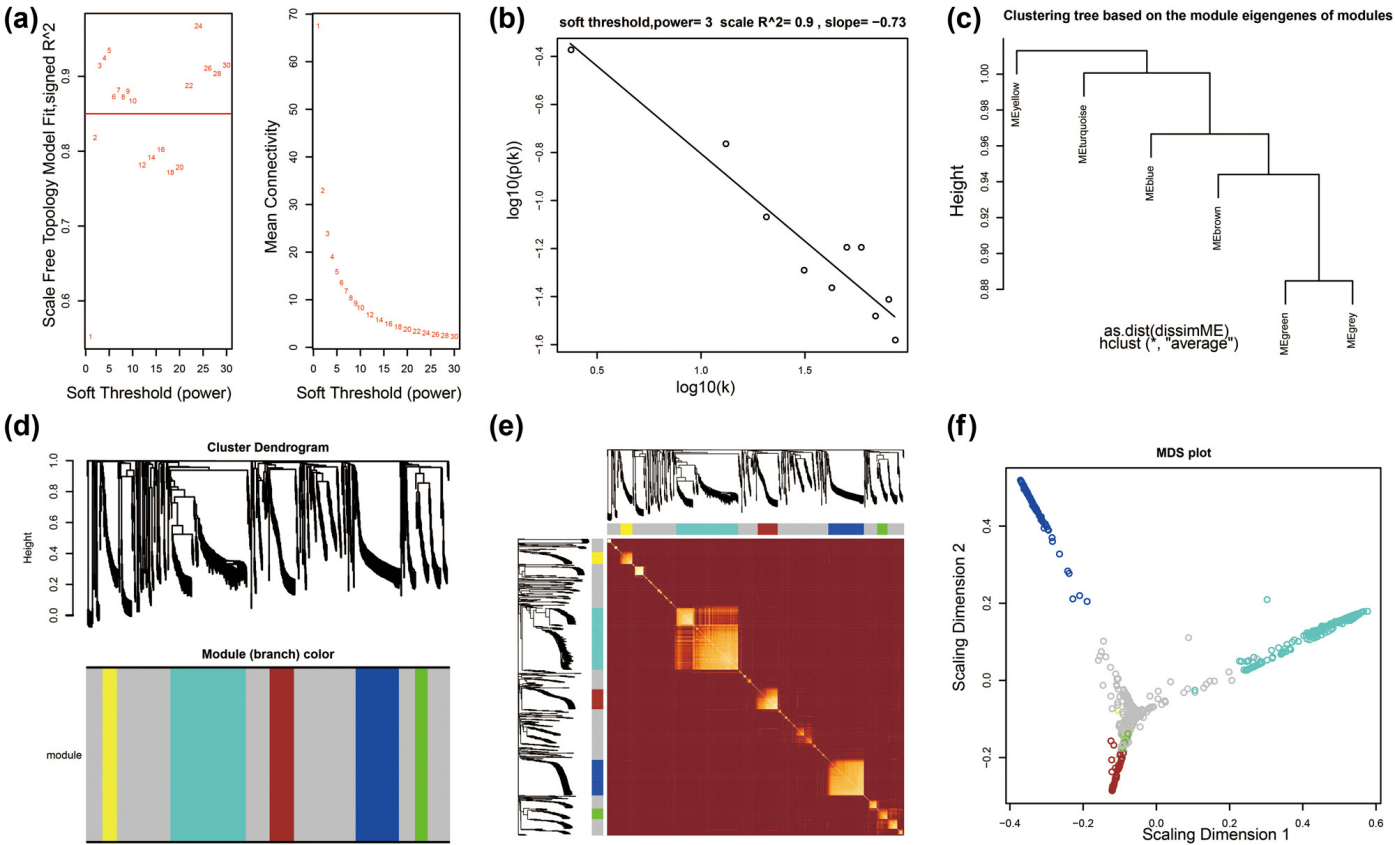
WGCNA analysis results of DEG: (a) soft thresholding; (b) scale-free plot; (c) clustering tree based on the module Eigen genes of modules; (d) cluster dendrogram; (e) topological overlap matrix (TOM) plot; and (f) multidimensional scaling plot.

**Figure 11:**
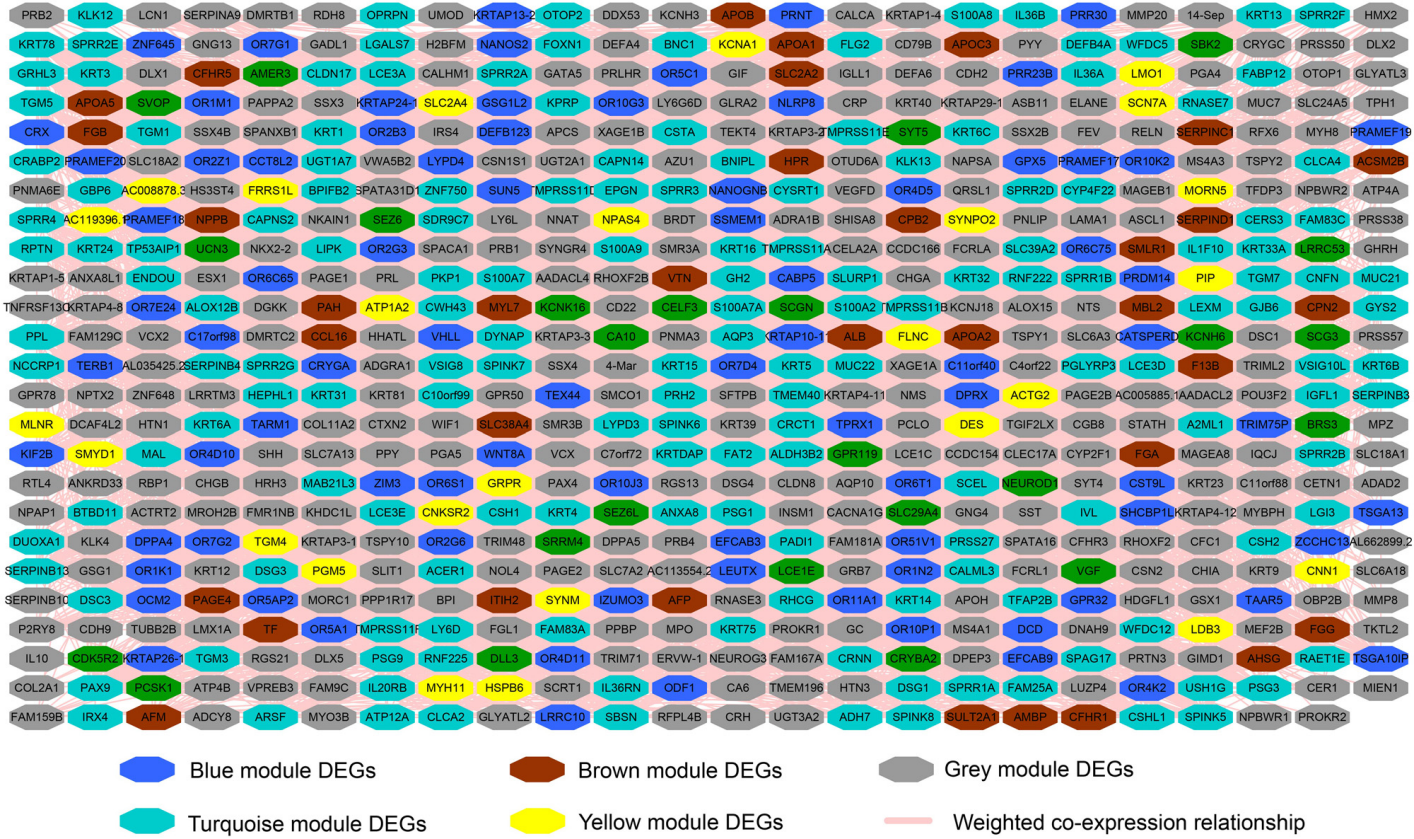
WGCNA interaction network of DEGs.

**Figure S2:**
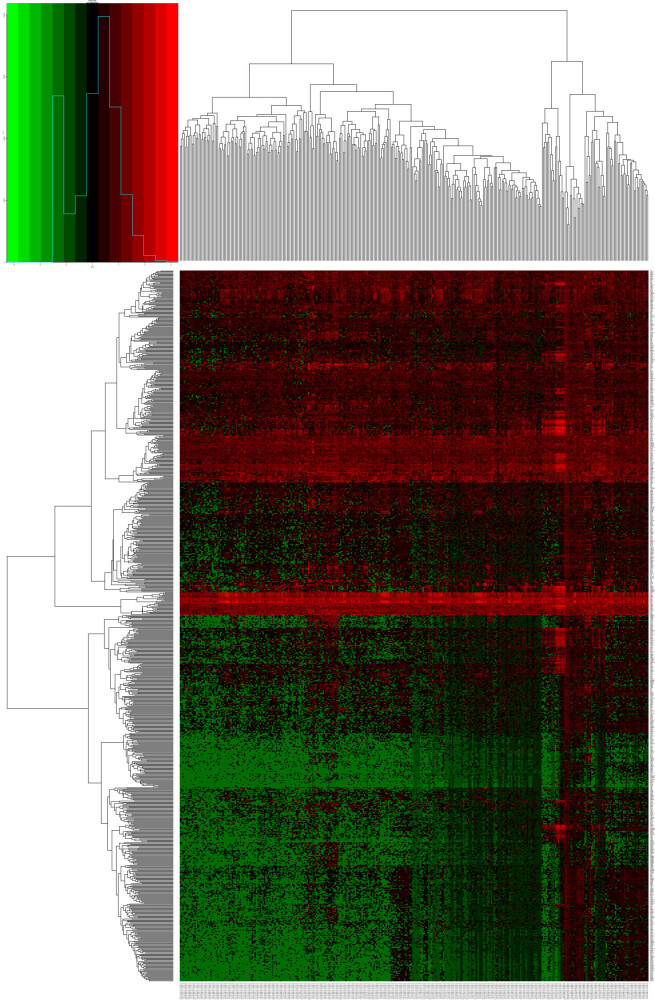
Heat map of DEGs between low- and high-risk score phenotypes of gastric cancer patients.

We screen for DEGs between high- and low-risk groups of GC patients, and 878 DEGs were generated, of which 623 were signally upregulated and 256 were signally downregulated (Figure 8, Figure S2 and Table S9). Survival analysis found that 83 DEGs were significantly associated with GC OS, among which the top three DEGs of significance were chorionic gonadotropin subunit beta 5 (CGB5), chorionic gonadotropin subunit beta 8 (CGB8), and secretin receptor (SCTR) (Figure 9a–d, Table S10). Then, we performed WGCNA and observed that these DEGs can be significantly divided into six modules: the top three modules are turquoise, blue, and brown modules, respectively (Figure 10a–f, Table S11). By analyzing the number of nodes of each DEG in the WGCNA network, we observed that the DEGs of the turquoise module have the highest degree, the highest degree value is 84, and there are three DEGs, namely cornulin, calmodulin-like 3, and cysteine-rich tail 1 (Figure 11, Table S12). These three genes may play the role of hub genes in this WGCNA network, especially in the turquoise module. GO term analysis revealed that these DEGs may be involved in G-protein-coupled receptor signaling pathway, cell differentiation, G-protein-coupled receptor activity, negative regulation of T-cell proliferation, cell–cell signaling, negative regulation of cytokine secretion involved in immune response, negative regulation of endothelial cell apoptotic process, opioid receptor signaling pathway, homophilic cell adhesion via plasma membrane adhesion molecules, negative regulation of nucleic acid-templated transcription, regulation of immune system process, retinoic acid metabolic process, negative regulation of endothelial cell apoptotic process, extracellular negative regulation of signal transduction, transcriptional activator activity, and RNA polymerase II transcription regulatory region sequence-specific binding (Table S13). KEGG suggested that these DEGs participated in the metabolism of xenobiotics by cytochrome P450, chemical carcinogenesis, and drug metabolism − cytochrome P450 (Table S13).

### 3.3 Immunomicroenvironment and ssGSEA analysis

**Figure 12:**
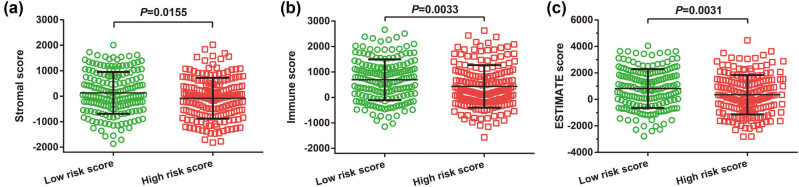
Results of tumor immune microenvironment analysis between low- and high-risk score phenotypes in GC: (a) Stromal score; (b) Immune score; and (c) ESTIMATE score.

**Figure 13:**
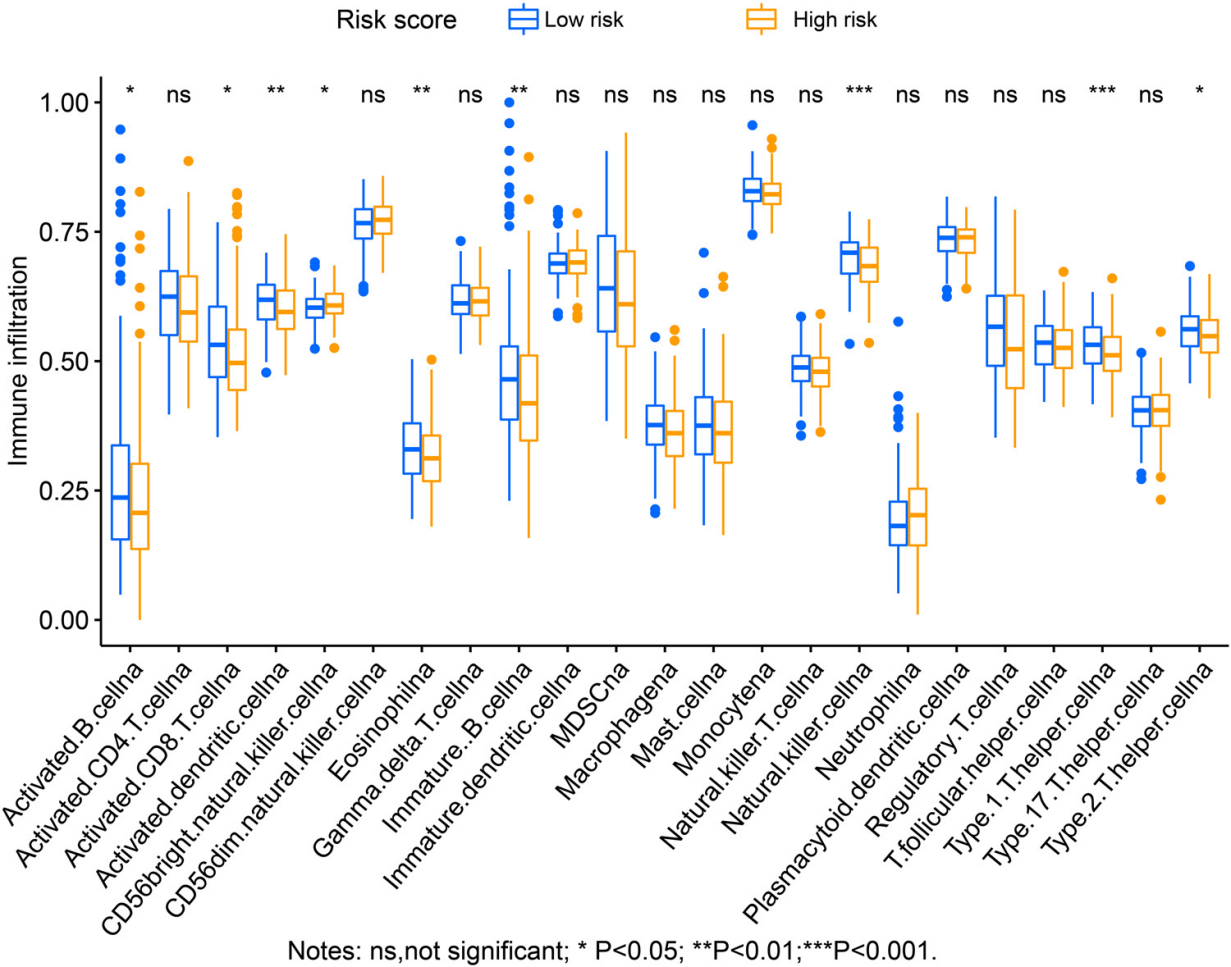
Immune infiltration analysis results of ssGSEA.

By analyzing the immune microenvironment in GC tumor tissues, we observed that three immune microenvironment scores were signally downregulated in tumor tissues of GC patients in the high-risk phenotype (Figure 12a–c). Through ssGSEA analysis, we compared the infiltration abundance of 23 immune cells and found that the infiltration abundance of 9 immune cells was notably downregulated in the high-risk score group (Figure 13).

## 4 Discussion

We all know that non-coding RNAs act an increasingly indispensable role in tumors. As the main component of the post-transcriptional RNA spliceosome, snRNA is involved in the processing of mRNA precursors. Through literature review, we did not find any related reports on snRNA in GC. In this study, we first downloaded the whole-genome RNA sequencing data set from the TCGA website. By extracting snRNAs data set, we compared the expression levels of snRNAs in tumor and adjacent non-tumor tissues. We found that a large number of snRNAs were differential expressions between tumor and adjacent non-tumor tissues. These differentially expressed snRNAs may lead to abnormal processing of mRNA precursors, resulting in dysregulation of GC-related gene transcription, which induces tumorigenesis and progression. Then, based on snRNA prognosis analysis, we obtained 14 snRNAs associated with GC prognosis and constructed a prognostic signature including 9 snRNAs. After a literature search, we found that among these nine snRNAs, only U1 has been reported in previous studies, and the remaining eight are novel and unreported snRNAs. Yin et al. found that U1 can mediate the binding of interacting RNA to chromatin, thereby affecting the function of RNA on chromatin [13]. Spraggon and Cartegni reviewed the physiological role of U1 in inhibiting polyadenylation in cells and suggested that this physiological role might be used in the treatment of cancers [32]. Another study has confirmed that the inhibition of U1 in Hela cells can signally increase the invasion and migration of cancer cells, while the opposite phenomenon was observed in Hela cell lines when U1 was overexpressed. At the same time, similar phenomena were observed in lung and breast cancer cell lines [33]. The study by Suzuki et al. found that U1 is frequently mutated in sonic hedgehogs, and the mutated U1 can significantly inhibit the tumor suppressor gene PTCH1 and activate the oncogenes GLI2 and CCND2. Their study concludes that U1 may be a potential therapeutic target for sonic hedgehogs [34].

Rahman et al. verified that U1 was significantly upregulated in Canine Melanoma using small RNA sequencing and quantitative reverse transcription-polymerase chain reaction [35]. Sadik et al. observed that U1 participates in immune regulation through TLR signaling pathway in the A549 lung cancer cell line, which may have anti-inflammatory effects [36]. Modification of U1 can significantly reduce the expression level of the human chorionic gonadotropin beta subunit and induce cervical cancer cell apoptosis [37]. Dong et al. observed that U1 was signally downregulated in lung cancer patients by analyzing the serum of lung cancer patients, and the ROC curve suggested that U1 may be a potential diagnostic biomarker for lung cancer [38]. High-frequency mutations of the third base of U1 snRNA have been reported in multiple cancers. The mutated U1 changes the splicing connection of the 5′ splicing site, resulting in changes in the splicing pattern of multiple genes, including some known oncogenes, resulting in the occurrence, development, and poor prognosis of cancers [39]. Cheng et al. overexpressed U1 in PC-12 cell line, and functional enrichment of DEGs screened by genome-wide expression microarray can enrich a large number of cancer-related signaling pathways. It was further inferred that U1 may activate a number of cancer-related functional mechanisms in adrenal pheochromocytoma [40]. U1 can also act as an adaptor to mediate drug resistance and regulate downstream gene expression levels in cancers [41,42,43]. By reviewing the above literature, we found that among the known snRNAs, U1 has been reported to play a variety of functions in cancers and can be used as a biomarker for various cancers. However, it has not been reported in GC. This study is the first to report that U1 can be used as a diagnostic and prognostic biomarker in GC. At the same time, it also explores its molecular mechanism, which provides a theoretical basis for the clinical application and functional mechanism exploration of U1 in GC. In the current study, we have found that U1 was signally downregulated in GC tumor tissues, which was consistent with previous reports. At the same time, it was previously found that U1 was related to the prognosis of CLL. We also observed that high expression of U1 was signally correlated with unfavorable prognosis in GC.

In terms of functional enrichment analysis, previous studies have reported that the physiological roles of snRNA are involved in alternative splicing, chromatin stabilization, and TLR signaling pathways. We also found that this prognostic signature snRNA could be significantly enriched in alternative splicing and TLR signaling pathways through functional enrichment analysis. In addition, we also enriched classical cancer-related signaling pathways such as Notch and PI3K. These results all suggest that snRNAs play an indispensable function in cancers. Since the previous literature has not conducted in-depth exploration and verification of snRNAs, our results still need to be further verified in the future.

There are some deficiencies in this study that need to be stated. First, all the results of this study are resulting from the functional enrichment analysis of the whole genome, which needs to be verified by further cell and animal experiments. Second, the survival analysis in the present study was a single-cohort analysis and lacked other multicenter validation cohorts. Despite the above shortcomings of this study, our research still includes the prognostic analysis of snRNAs to the functional enrichment analysis of various approaches and preliminarily clarified the clinical significance and potential biological molecular mechanism of snRNAs in GC. These results can provide some theoretical support and a basis for future research.

## 5 Conclusion

In the present study, we obtained 14 prognostic snRNA markers significantly associated with GC OS and also constructed a prognostic signature containing nine prognostic snRNAs. Both survivalROC and nomogram model suggest that this prognostic signature can be a good indicator for predicting OS in GC patients. Molecular mechanism exploration found that this prognostic signature is involved in classical cancer-related signaling pathways such as Notch, PI3KAKT, TLR signaling pathways, and biological processes such as cell cycle, cell proliferation, and cell adhesion that affect the biological phenotype of cancer cells. Immune-related analyses also suggested significant differences in the tumor immune microenvironment and immune cell infiltration between high- and low-score patients classified according to this prognostic signature. As all the conclusions of this study were generated from the bioinformatics analysis and clinical analysis of whole-genome RNA sequencing data, all the results still need to be further verified in future studies.

## Supplementary Material

Supplementary Table
